# Antenatally diagnosed ovarian cyst with torsion managed laparoscopically

**DOI:** 10.4103/0971-9261.42571

**Published:** 2008

**Authors:** A. K. Singal, K. G. Vignesh, Sarah Paul, John Matthai

**Affiliations:** MGM Medical College, Kamothe, Navi Mumbai, Maharashtra, India; 1 Department of General Surgery, PSG Institute of Medical Sciences, Coimbatore, Tamilnadu, India; 2 Department of Pediatrics, PSG Institute of Medical Sciences, Coimbatore, Tamilnadu, India

**Keywords:** Laparoscopy, neonate, ovarian cyst

## Abstract

Ovarian cyst are the most common intra-abdominal cyst in female neonate. With the help of ultrasound one can make an antenatal diagnosis. We present one such neonate, she was managed by laparoscopic excision. We conclude that neonatal laparoscopy is technically feasible for management of such cysts.

## INTRODUCTION

With the advent and routine availability of quality antenatal ultrasound, ovarian cysts in the fetus are diagnosed more frequently these days. Ovarian cysts are the most common intraabdominal cysts in the female neonate. Most of these cysts regress in the first 6 months of life. However, large cysts and complex cysts may require active management.

In the present era of minimally invasive surgery, ovarian cysts are increasingly being managed laparoscopically.[1–4]

We report a neonate with antenatally diagnosed ovarian cyst that was managed laparoscopically.

## CASE REPORT

**Figure 1:**
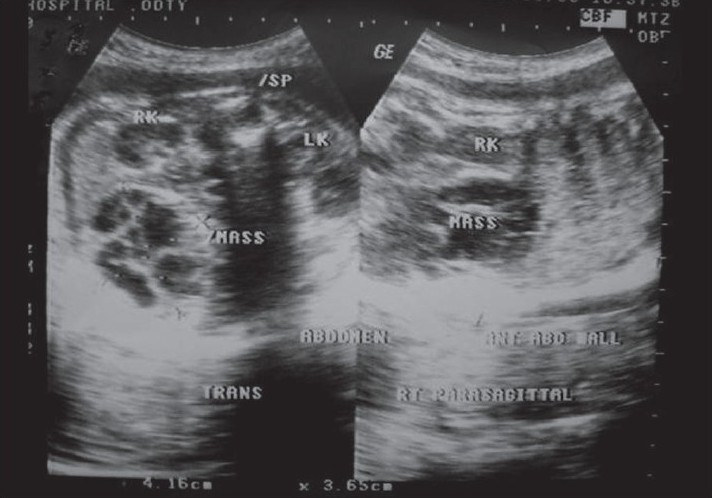
Ultrasound showing complex ovarian cyst

A 7-day-old female neonate was referred to our hospital with an antenatal diagnosis of an intraabdominal cyst. The cyst had multiple septae and was 4 cm in size. She was asymptomatic. A repeat USG showed a complex ovarian cyst with a size of 4.5 cm that occupied the right iliac fossa [Figure 1]. The other ovary was not visualized.

**Figure 2:**
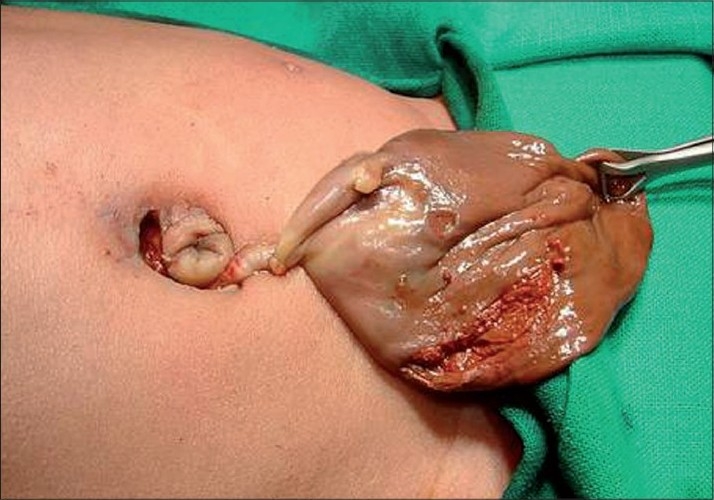
Cyst delivered outside the abdomen

The child was taken up for laparoscopy. A 5-mm trocar was inserted through the umbilical route via open technique. Pneumoperitoneum was established at a pressure of 6 cm. Laparoscopy revealed a large chocolate-colored right ovarian cyst with the torsion of its pedicle. The opposite ovary was normal. A 3-mm port was placed in the left iliac fossa via the interpenetration technique. The cyst was held onto the anterior abdominal wall with a Maryland dissector and decompressed with a transcutaneous wide-bore needle. The contents were altered blood and clots. The umbilical incision was enlarged to 1 cm and the cyst was delivered outside. With gentle rocking movements, the whole cyst could be delivered easily through the small umbilical incision [Figure 2]. The pedicle was transfixed with vicryl suture and the specimen was removed. A thorough wash was given to remove any spilled-out material. Umbilical fascia was closed with monocryl and the skin was closed with subcuticular stitches.

Postoperatively, the child was allowed orally after 24 h and was discharged uneventfully on the third day. She has remained asymptomatic on follow-up after 1 month.

## DISCUSSION

Neonatal ovarian cysts result from the abnormal stimulation of mature ovarian follicles by maternal gonadotrophins in the fetal stage. These are more common in neonates whose mothers had abnormally high levels of HCG such as diabetes, maternal isoimmunization or in premature babies, where the ovaries are highly sensitive to HCG stimulation.[2,5,6]

With the increased use of prenatal USG, the detection rate for these cysts has increased considerably. Upto 34% of the fetuses may have sonographically detectable cysts antenatally.[7] Management is not required for all of these cysts. It has been suggested that after delivery, as the anterior pituitary starts the negative-biofeedback mechanism, the abnormal gonadotrophin secretion is discontinued; many of these cysts regress spontaneously, although regression may take up to 10 months.[5,6]

According to Nussbaum's classification, the neonatal ovarian cysts can be classified into simple or uncomplicated (completely anechoic) and complex or complicated (fluid debris level, clot, septae, and echogenic wall) cysts, suggesting torsion.[7]

Untreated ovarian cysts can lead to complications such as hemorrhage, rupture and torsion. Torsion is the most common complication of an untreated ovarian cyst, and it generally occurs in large cysts, although it has also been reported in smaller cysts.[6,7]

Torsion has been reported to occur antenatally in most of the cases in literature and was also seen in the present case.[6,7]

Simple cysts are a result of benign stimulation and can be observed for regression if less than 4 cm in size. Given the propensity for torsion in larger cysts, surgical therapy is recommended. Surgery is also recommended in complex cysts as these are mostly the ones with torsion. Second, neoplasm can not be conclusively ruled out. Neoplasms have been reported even in 30-week-old fetuses.[2,5,6,8]

In the present era, most of these cysts can be managed laparoscopically. It has been shown to be safe even in neonates and has all the advantages of a minimally invasive approach.[1,2,4] As the neonatal ovary has a long pedicle, the cyst after decompression can be easily delivered via the umbilicus and ovariectomy can be performed. Laparoscopy offers the added advantages in case the diagnosis is not sure. In addition, the opposite ovary can be observed clearly.

In conclusion, ovarian cysts that require surgical procedure can be easily managed via laparoscopic approach.
